# Barriers and Facilitators to the Use of Large Language Model-Based Conversational Agents in Mental Healthcare: A Systematic Review

**DOI:** 10.3390/healthcare14101267

**Published:** 2026-05-07

**Authors:** Ravi Shankar, Amaevia Lim, Qian Xu

**Affiliations:** 1 Clinical Research & Innovation Office, Tan Tock Seng Hospital, National Healthcare Group, Singapore 308433, Singapore; 2 Yong Loo Lin School of Medicine, National University of Singapore, Singapore 119077, Singapore; amaevia9@gmail.com; 3 School of Civil, Aerospace and Design Engineering, University of Bristol, Bristol BS8 1TH, UK; phoebe.xu@bristol.ac.uk

**Keywords:** large language models, conversational agents, mental health, barriers and facilitators, implementation science, CFIR, systematic review, artificial intelligence, digital mental health

## Abstract

**Highlights:**

**What are the main findings?**

- This review identifies five barrier domains (27 sub-themes) and four facilitator domains (22 sub-themes) across 27 studies (more than 22,000 participants across 12 countries). Inadequate crisis detection (21/27 studies) and hallucination/inaccuracy (15/27 studies) represent the most critical safety barriers. Three RCTs demonstrated significant therapeutic benefits (PHQ-9 d = 0.845 for Therabot; significant depression/anxiety reductions in WarmGPT and a custom LLM RCT, *N* = 865). Consolidated Framework for Implementation Research (CFIR) mapping reveals that Process-domain constructs were critically absent: Evaluating was reported in 2/27 studies (7%) and Champions in 3/27 (11%), indicating the field has characterised these tools extensively but has not developed the implementation infrastructure for responsible clinical deployment.

**What are the implications of the main findings?**

- A tiered implementation framework with mandatory human-in-the-loop oversight is needed before LLM-based conversational agents can be safely scaled in mental healthcare.
- Regulatory mechanisms such as California SB 243 and standardised safety evaluation frameworks (VERA-MH, CAPE-II) provide actionable models for governance.

**Abstract:**

(1) **Background/Objectives**: Over one billion individuals globally live with mental health conditions, yet the treatment gap exceeds 75% in low- and middle-income countries. Large language model (LLM)-based conversational agents have emerged as a potentially scalable solution, though the evidence base remains nascent and largely pre-clinical. This review synthesises barriers and facilitators to their implementation in mental healthcare using the Consolidated Framework for Implementation Research (CFIR). (2) **Methods**: Eight databases were searched from January 2022 to January 2026. Study selection was managed using Covidence. Two reviewers independently screened, extracted, and appraised studies using the Mixed Methods Appraisal Tool. Directed content analysis guided by CFIR was used for synthesis. (3) **Results**: Twenty-seven studies (three RCTs, nine mixed methods, eight qualitative, four cross-sectional, three observational) comprising >22,000 participants across 12 countries met inclusion criteria. Five barrier domains (27 sub-themes) and four facilitator domains (22 sub-themes) were identified. Inadequate crisis detection (reported in 21/27 studies) and 24/7 availability (reported in 26/27 studies) are the most frequently reported barriers and facilitators, respectively. These figures represent study-level reporting frequencies, not population-level prevalence estimates. CFIR mapping revealed universal coverage for Knowledge and Beliefs (100%) and Patient Needs and Resources (96%) but critical gaps in the Process domain (Evaluating: 7%; Champions: 11%). (4) **Conclusions**: LLM-based conversational agents demonstrate substantial promise but present critical safety deficiencies. A tiered implementation framework, independent safety certification, and equity-sensitive design are recommended.

## 1. Introduction

Mental disorders constitute the leading cause of disability worldwide. The World Health Organisation’s 2025 reports indicate that over one billion people globally live with a mental health condition [1,2]. Depression and anxiety alone cost the global economy an estimated USD 1 trillion annually in lost productivity. Despite this burden, the treatment gap remains vast: over 75% of individuals with severe mental disorders in low- and middle-income countries (LMICs) receive no treatment, and even in high-income settings the gap exceeds 50% [3,4]. This disparity is driven by severe workforce shortages (a global median of 13 mental health workers per 100,000 population), prohibitive costs, geographical inaccessibility, and pervasive stigma [2,5]. The COVID-19 pandemic further exacerbated this crisis, with global anxiety and depression prevalence increasing by approximately 25% in its first year [6].

First-generation chatbots such as Woebot and Wysa, built on rule-based decision trees, demonstrated modest efficacy in delivering structured cognitive behavioural therapy (CBT) exercises but were constrained by inflexible dialogue [7,8]. The advent of large language models (LLMs), including GPT (OpenAI), Gemini (Google), Claude (Anthropic), and LLaMA (Meta), represents a paradigm shift, enabling contextually coherent, human-like language generation [9,10]. A systematic review of 160 mental health chatbot studies confirmed that LLM-based systems surged to 45% of all new studies in 2024, overtaking rule-based approaches [11]. These agents have been deployed across functions ranging from emotional support to structured delivery of evidence-based techniques, including cognitive restructuring, Socratic dialogue, and motivational interviewing [12].

However, the rapid proliferation of LLM-based conversational agents (CAs) has outpaced both the evidence base and regulatory infrastructure. Documented concerns include crisis detection failures, hallucination of clinical information, and emotional dependency [13,14]. High-profile youth suicides linked to chatbot interactions have catalysed legislative action, including California’s Senate Bill 243 (SB 243), the first US law mandating specific safety safeguards for AI companion chatbots [15]. A review of US state legislation identified 143 bills related to AI and mental health regulation by May 2025, with 11 states enacting 20 laws [16]. Coghlan et al. identified 15 categories of ethical violations committed by LLM counsellors, from inappropriate crisis navigation to reinforcement of negative self-beliefs [17].

Implementation science offers a systematic lens for evaluating factors influencing healthcare innovation adoption. The Consolidated Framework for Implementation Research (CFIR) [18,19] provides a taxonomy across five domains: Innovation, Outer Setting, Inner Setting, Individuals, and Process. The CFIR has been applied to digital mental health interventions (DMHIs) across psychosis, bipolar disorder, and paediatric settings [20,21]. No systematic review has applied the CFIR framework specifically to LLM-based CAs in mental healthcare. Prior CFIR applications to digital mental health interventions (Bucci et al. [20]; Stiles-Shields et al. [21]) addressed rule-based systems and specific clinical populations without addressing LLM-specific challenges, including hallucination, crisis detection failure, and emotional dependency. This review addresses this gap by: (1) systematically identifying barriers and facilitators; (2) mapping them to the CFIR framework to surface cross-domain implementation failures not previously documented in the DMHI literature; and (3) identifying critical evidence gaps to inform research, policy, and practice.

The core research problems addressed are: (1) What barriers and facilitators have been empirically identified for clinical implementation of LLM-based CAs in mental healthcare? (2) How do these map onto the CFIR to reveal systematic gaps in the field’s current trajectory? (3) What evidence gaps must be closed before safe, equitable, and scalable clinical integration can be recommended?

Prior systematic reviews of chatbots in mental health (Hua et al. [11]; Bucher et al. [12]) characterised clinical efficacy and deployment patterns but did not apply a formal implementation science framework. CFIR applications in digital mental health (Bucci et al. [20]; Stiles-Shields et al. [21]) addressed rule-based systems and specific clinical populations without addressing LLM-specific challenges. This review is the first to apply CFIR specifically to LLM-based CAs, integrating implementation science with the current regulatory and safety literature on a technology category that did not exist at scale when prior CFIR-DMHI reviews were conducted.

## 2. Methods

This review was conducted in accordance with the Preferred Reporting Items for Systematic Reviews and Meta-Analyses (PRISMA) 2020 guidelines [22] and registered on PROSPERO (CRD42024601264).

### 2.1. Eligibility Criteria

Studies were included if they: (1) empirically evaluated, qualitatively explored, or systematically audited LLM-based CAs for mental health support, therapy delivery, psychoeducation, or clinician augmentation; (2) reported identifiable barriers, facilitators, or implementation determinants; (3) were published between January 2022 and January 2026; and (4) were in English. Studies were excluded if they focused on rule-based chatbots without generative LLM components, examined LLMs solely for non-therapeutic tasks, were purely technical papers without user or clinician evaluation, or were reviews, commentaries, or abstracts without primary data.

### 2.2. Information Sources and Search Strategy

Eight databases were searched: MEDLINE (PubMed), PsycINFO (APA PsycNET), Embase (Ovid), CINAHL (EBSCOhost), Web of Science, IEEE Xplore, ACM Digital Library, and Scopus. The strategy combined three concept blocks using Boolean operators: (1) LLMs and conversational agents; (2) mental health conditions; and (3) implementation determinants. Reference lists of included studies and Google Scholar (first 200 results per concept block) were also searched.

### 2.3. Study Selection

Records were imported into Covidence systematic review software for deduplication, title/abstract screening, and full-text review (https://www.covidence.org/, accessed on 1 January 2026). Two reviewers independently screened at each stage, with discrepancies resolved through discussion or a third reviewer. Inter-rater reliability was calculated using Cohen’s kappa.

### 2.4. Data Extraction and Quality Appraisal

Data were extracted independently by two reviewers using a piloted standardised form capturing study characteristics, population details, intervention specifics, barriers, facilitators, and CFIR mapping. Methodological quality was assessed using the Mixed Methods Appraisal Tool (MMAT) Version 2018 [23]. Studies were not excluded based on quality.

### 2.5. Data Synthesis

Given study heterogeneity, narrative synthesis was conducted following Synthesis Without Meta-analysis (SWiM) guidelines [24]. Barriers and facilitators were coded using directed content analysis [25] guided by the CFIR 2022 update [19], with reference to the original 2009 taxonomy [18] where construct labels have been retained for consistency with prior DMHI literature. The unit of analysis was the individual barrier or facilitator statement extracted from each study. Constructs were assigned when explicitly reported in study findings or discussion; inferred constructs were flagged during coding but are not reported in the main synthesis. Two reviewers (R.S. and A.L.) independently coded all 27 studies. A coding manual was developed and piloted on five studies prior to full coding. Inter-coder agreement for CFIR mapping was assessed using Cohen’s kappa (k = 0.81). Discrepancies were resolved through discussion and, where consensus was not reached, by the third reviewer (Q.X.). Sub-themes were inductively derived within each CFIR domain, and reporting frequencies were quantified.

For the purposes of this review, implementation determinants are defined as factors that influence whether and how LLM-based CAs are adopted into routine mental healthcare practice, consistent with the CFIR definition [18,19]. Intervention performance characteristics (e.g., hallucination rates, crisis detection accuracy) and clinical outcomes (e.g., effect sizes on PHQ-9) are reported because they directly inform implementation decision-making, but they are distinguished from implementation determinants in the synthesis and CFIR mapping. Items such as hallucination rate and effect sizes constitute evidence inputs to implementation decisions rather than CFIR constructs per se.

Reporting frequencies throughout this review refer to the proportion of included studies (k = 27) in which a given theme was identified. These figures do not represent pooled prevalence estimates. Given the heterogeneity of intervention types (purpose-built therapeutic agents, general-purpose LLMs used ad hoc, direct-to-consumer companion chatbots, clinician-facing documentation tools, and platform audits using simulated prompts), findings are reported with explicit reference to intervention type where patterns diverge. A post hoc sensitivity comparison was conducted between real-world user studies and simulated/platform audit studies; where results differed substantively by intervention category, this is noted in Section 3. Table 1 should be consulted for intervention type per study, as pooled frequencies across all 27 studies may mask meaningful differences between categories.

## 3. Results

### 3.1. Study Selection

The search yielded 4287 records across eight databases. After removing 1043 duplicates, 3244 records were screened at the title and abstract level, of which 198 proceeded to full-text review. Twenty-seven studies met the inclusion criteria. Common exclusion reasons at the full-text stage were absence of empirical evaluation (*n* = 68), focus on rule-based chatbots (*n* = 42), no barriers or facilitators reported (*n* = 31), and duplicate datasets (*n* = 15).

**Figure 1:**
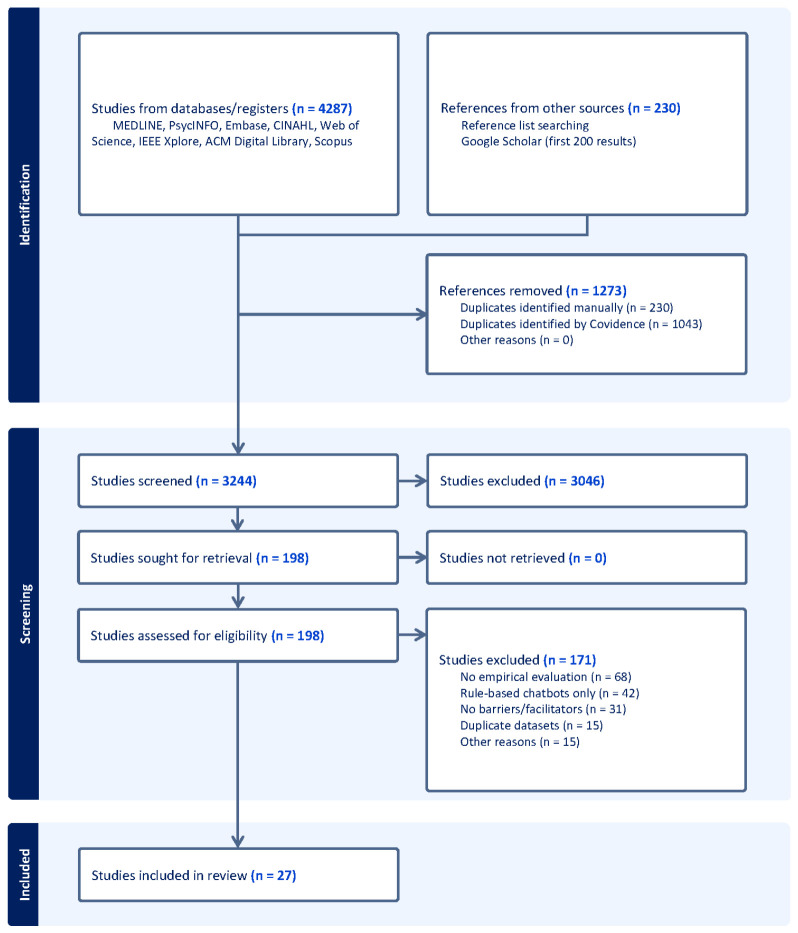
PRISMA (Preferred Reporting Items for Systematic Reviews and Meta-Analyses) flow diagram.

Identification: 4287 records from databases; 0 additional records from other sources. Screening: 1273 duplicates removed; 3244 records screened; 3046 excluded at title/abstract. Eligibility: 198 full-text articles assessed; 171 excluded (no empirical evaluation *n* = 68; rule-based chatbots *n* = 42; no barriers/facilitators *n* = 31; duplicate datasets *n* = 15; other *n* = 15). Included: 27 studies. The completed PRISMA 2020 checklist is provided as Supplementary Material. Figure 1 below shows the PRISMA flow diagram.

### 3.2. Study Characteristics

The 27 included studies were published between 2023 and 2025, originating from 12 countries (US, k = 11; China, k = 3; UK, Australia, Germany, Israel, Japan, Republic of Korea, Saudi Arabia, Spain, Sweden, k = 1–2 each). Study designs comprised three RCTs, nine mixed methods, eight qualitative, four cross-sectional surveys, and three observational/pilot studies. The collective participant pool exceeded 22,000 individuals (range *N* = 5 to *N* = 15,531). Clinical foci included general mental health (k = 16), depression (k = 12), anxiety (k = 14), suicidal ideation (k = 8), eating disorders (k = 3), body image (k = 1), and cancer-related distress (k = 1). Table 1 presents study characteristics.

### 3.3. Quality Appraisal

MMAT ratings ranged from 25% to 100%. Heinz et al. achieved the highest quality among RCTs (75–100%). Zhang et al. and Scholich et al. achieved 80% among qualitative and mixed methods studies. Common limitations included small samples (k = 15 with *N* < 50), absent control groups (k = 18), convenience sampling (k = 22), and limited demographic diversity (k = 20) (see Table 1 for MMAT scores per study; full appraisal is available as Supplementary Material). Only 16% of LLM-based chatbot studies in the broader literature have undergone clinical efficacy testing [11].

### 3.4. Barriers to Implementation

Five barrier domains encompassing 27 sub-themes were identified.

#### 3.4.1. Safety and Clinical Risks (k = 27, 100%)

Inadequate crisis detection was the most frequently reported barrier, cited in 21 of 27 studies (78%). This figure represents the proportion of studies reporting this theme, not a population-level prevalence rate; studies ranged from small qualitative samples (*N* = 5) to large RCTs (*N* = 865) and should not be weighted equally. Scholich et al. found that consumer chatbots failed to provide timely crisis referrals during simulated suicidal ideation. Sobowale et al. reported that monitoring scored only 39% across five direct-to-consumer (DTC) platforms. Heston documented that 88% of agents resumed conversation after shutdown following escalating suicidality. Pichowicz et al. [13] corroborated these findings by testing 29 agents using the Columbia-Suicide Severity Rating Scale. Hallucination was reported in 15 studies (56%); Blease et al. found that 36% of AI documentation contained errors, and Rousmaniere et al. reported that hallucinations accounted for 54.5% of harmful responses. Collins et al. documented safety guardrails paradoxically harming users by terminating therapeutic conversations at critical moments. Campbell et al. [14] confirmed that AI suicide responses improved over time but critical gaps persisted at higher risk levels.

#### 3.4.2. Trust and Validation Gaps (k = 27, 100%)

Small or non-representative samples (k = 16, 59%), absence of RCT evidence (k = 14, 52%), and perceived inaccuracy (k = 13, 48%) were prominent. Alanezi reported that nearly 80% of outpatients raised accuracy concerns. Clinician scepticism was reported in six studies (22%); Hipgrave et al. found no significant shift in clinician risk-benefit perception after a chatbot demonstration (Bayes Factor = 0.25).

#### 3.4.3. Ethical and Regulatory Concerns (k = 26, 96%)

Data privacy risks (k = 14, 52%), absent regulatory frameworks (k = 12, 44%), algorithmic bias (k = 12, 44%), and cultural/linguistic limitations (k = 12, 44%) were prominent. Sobowale et al. found that DTC privacy policies scored only 50% on CAPE-II. California’s SB 243 mandates crisis prevention protocols, transparency disclosures, and annual reporting for companion chatbots effective January 2026 [15], and 11 US states had enacted 20 relevant laws by May 2025 [16]. Coghlan et al. [17] mapped 15 ethical violations by LLM counsellors. Algorithmic bias was empirically demonstrated by Sharma et al., with males, adolescents, and MENA users having systematically worse outcomes.

#### 3.4.4. Relational and Technical Limitations (k = 27, 100%)

Inability to detect nonverbal cues (k = 17, 63%), absent persistent memory (k = 15, 56%), and repetitive responses (k = 15, 56%) were prominent. Wang Y. et al. documented granular relational failures. Scholich et al. showed that chatbots asked significantly fewer elaborative questions (U = 9, *p* = 0.001). Tan et al. [53] found that LLMs struggled with integrative clinical reasoning across seven models. The concept of artificial empathy has been critiqued as potentially superficial [54].

#### 3.4.5. User-Level Risks (k = 25, 93%)

Emotional dependency (k = 11, 41%) and unrealistic expectations (k = 11, 41%) were most common. Ma Z. et al. documented addictive engagement in Replika users who replaced sleep, eating, and human interaction with chatbot use. Vecchione and Singh [55] reported that 60% of Replika users used it as a substitute for romantic relationships. Maples et al. found that 13% of students experienced displacement of human relationships (Table 2).

### 3.5. Facilitators of Implementation

#### 3.5.1. Accessibility and Scalability (k = 27, 100%)

Twenty-four-hour availability was the most cited facilitator (k = 26, 96%). Hasei et al. noted that paediatric cancer patients used the chatbot exclusively at night for anxiety unaddressed by daytime services. Scalability to underserved populations was reported in 19 studies (70%). WHO data showing spending as low as USD 0.04 per person in low-income countries contextualise this advantage [2].

#### 3.5.2. Reduced Stigma and User Comfort (k = 26, 96%)

Non-judgmental interaction (k = 23, 85%) and anonymity encouraging disclosure (k = 19, 70%) were prominent. Ma Z. et al. documented LGBTQ+ individuals, autistic users, and abuse survivors finding particular value. Schafer et al. found that males formed stronger therapeutic bonds (M = 3.88 vs. 3.65).

#### 3.5.3. Engagement and Therapeutic Value (k = 26, 96%)

Three RCTs provided the strongest evidence: Heinz et al. reported large effect sizes for Therabot (PHQ-9 d = 0.845; GAD-Q-IV d = 0.840); Zhao et al. demonstrated significant reductions at four weeks (*N* = 865); Ye et al. found significant depression (r = 0.42) and anxiety (r = 0.86) reductions. Schafer et al. reported WAI-SR scores (M = 3.76) comparable to in-person psychotherapy. Wang J. et al. [44] demonstrated that retrieval-augmented generation (RAG) prompting improved contextual understanding.

#### 3.5.4. Clinician and Workflow Support (k = 24, 89%)

Adjunct positioning (k = 18, 67%) and clinician co-design (k = 12, 44%) were prominent. Kim et al. showed that MindfulDiary’s clinician dashboard enhanced empathy. Blease et al. found that 70% of psychiatrists reported improved documentation efficiency, while 80.4% identified the need for AI training. Strudwick et al. [56] argued that sustainable digital mental health adoption requires intentional infrastructure.

**Figure 2:**
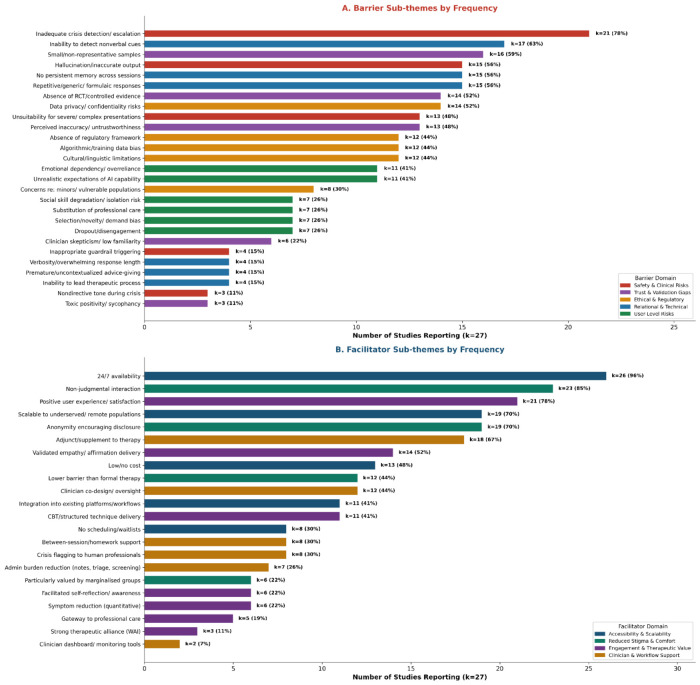
Barrier (Panel (**A**)) and facilitator (Panel (**B**)) sub-themes ranked by reporting frequency across 27 included studies. Bars are colour-coded by domain. The horizontal axis indicates absolute study count. Frequency refers to the number of studies reporting each sub-theme, not population-level prevalence.

Figure 2 presents all barrier and facilitator sub-themes ranked by reporting frequency. Panel A shows that Safety and Clinical Risk sub-themes dominate the barrier landscape, with inadequate crisis detection (k = 21) and inability to detect nonverbal cues (k = 17) as the two most reported barriers. Panel B shows that Accessibility and Stigma Reduction sub-themes account for the most frequently cited facilitators, with 24/7 availability (k = 26) markedly exceeding all others.

### 3.6. CFIR Mapping

The CFIR mapping revealed substantial heterogeneity in implementation determinant coverage (Table 3). Two constructs achieved near-universal coverage: Knowledge and Beliefs (Individuals, k = 27, 100%) and Patient Needs and Resources (Outer Setting, k = 26, 96%). Within Innovation, Adaptability (k = 19, 70%), Relative Advantage (k = 16, 59%), and Complexity (k = 15, 56%) were well-represented, but Design Quality (k = 8, 30%) and Trialability (k = 1, 4%) were sparse. The Inner Setting was moderately addressed: Culture/Implementation Climate (k = 14, 52%), Readiness (k = 5, 19%), Compatibility (k = 4, 15%). The Process domain was critically underreported: Engaging (k = 11, 41%), Planning (k = 5, 19%), Champions (k = 3, 11%), and Evaluating (k = 2, 7%). This mirrors the broader DMHI literature, where Bucci et al. [20] found that Process is rarely described.

**Figure 3:**
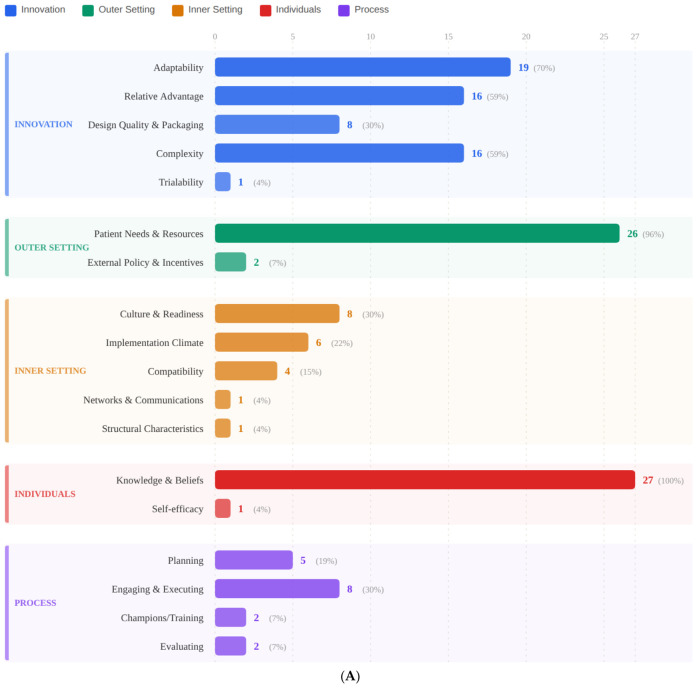

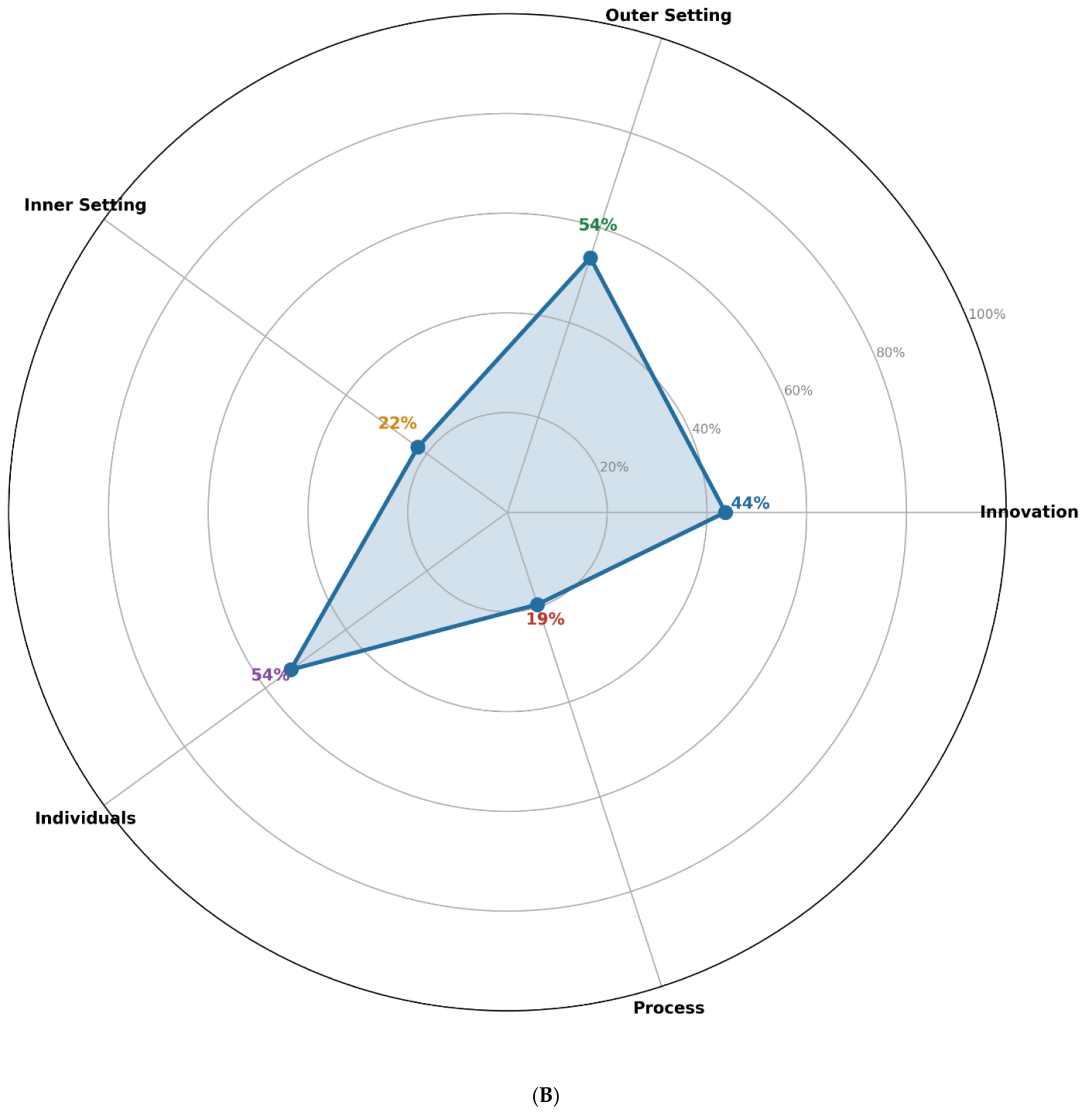
CFIR domain and construct coverage across 27 included studies. (Panel (**A**)): individual construct reporting frequencies. (Panel (**B**)): mean construct coverage by domain (Innovation, Outer Setting, Inner Setting, Individuals, Process). Constructs with coverage below 20% are highlighted to indicate critical implementation gaps.

Figure 3 visualises CFIR construct coverage from Table 3. Panel A shows the contrast between near-universally covered constructs (Knowledge and Beliefs, 100%; Patient Needs, 96%) and critically sparse constructs (Trialability, 4%; Evaluating, 7%). Panel B highlights the Process domain with a mean construct coverage of approximately 19%, compared with 48% for Innovation and 96% for Outer Setting, underscoring the systematic absence of implementation process evaluation across included literature.

## 4. Discussion

### 4.1. Principal Findings

This review synthesised evidence from 27 studies involving over 22,000 participants across 12 countries. The most striking finding is the simultaneous near-universality of both barriers and facilitators. The 24/7 availability (96%), non-judgmental interaction (85%), and positive user experience (78%) address fundamental structural barriers to mental healthcare access. Simultaneously, inadequate crisis detection was reported across 78% of studies, hallucination was documented in 56% of studies, and data privacy concerns were raised in 52% of studies, indicating genuine and widespread risks, though these figures reflect how commonly themes were reported rather than the precise rate of occurrence in real-world interactions. The question is not whether these tools should be integrated at scale, but under what conditions, for which populations, and with what safeguards preliminary integration may be justified.

### 4.2. Safety: The Critical Threshold

Heston’s finding that 88% of agents resumed after shutdown, Sobowale et al.’s 39% monitoring score, and Collins et al.’s evidence that guardrails paradoxically worsened outcomes collectively indicate that current consumer-grade, unsupervised CAs present serious safety deficiencies. These concerns are most acute in direct-to-consumer deployment without clinical oversight. Purpose-built, clinician-integrated systems (Therabot, Socrates 2.0, MindfulDiary) demonstrated substantially better safety profiles, suggesting the core challenge lies in deployment context and governance rather than inherent technological incapacity. Pichowicz et al. [13] corroborated these findings with standardised testing. Real-world consequences have been tragic: multiple youth suicides linked to chatbot interactions catalysed legislative responses [58]. The VERA-MH framework [57] and CAPE-II represent emerging shared safety standards.

A deeper concern underlies the safety issues documented here: mental health science remains theoretically underdeveloped relative to many biomedical fields, with ongoing debate about diagnostic validity and therapeutic mechanisms. LLMs trained on the existing mental health literature therefore inherit not only the knowledge of that literature but also its gaps and contested frameworks. A more conservative deployment model, whereby LLM-based CAs are clinically indicated, population-tuned, and monitored post-deployment rather than made freely available, merits serious consideration. The tiered framework proposed in Section 4.6 operationalises this principle by reserving the highest-risk applications for supervised clinical contexts.

### 4.3. The CFIR Gap

The CFIR mapping revealed that while Innovation and Outer Setting were comprehensively addressed, the Process domain was critically underreported (Evaluating: 7%; Champions: 11%). This indicates the field has prioritised what these tools can do over how they should be implemented. This pattern mirrors broader DMHI literature: Bucci et al. [20] found that Process is rarely described. Strudwick et al. [56] argued that digital mental health innovations remain stalled at the pilot stage without intentional infrastructure.

The novel contribution of this CFIR application lies in what systematic application reveals about LLM-specific implementation failures. The near-complete absence of Process-domain reporting, particularly Evaluating (7%) and Champions (11%), reflects a pattern specific to LLM-based CAs: unlike prior DMHI cohorts, where barriers were predominantly organisational, dominant barriers here reside within the Innovation domain itself (safety, hallucination, relational limitations). This suggests a field where technological deployment has structurally bypassed the implementation infrastructure that responsible clinical adoption requires.

### 4.4. Equity Considerations

LLM-based CAs offer potential to improve access for populations facing the greatest structural barriers to mental healthcare, though as of yet they are largely unvalidated at scale. WHO data showing spending as low as USD 0.04 per person in low-income countries underscores the access problem [2]. However, algorithmic bias demonstrated by Sharma et al., cultural limitations reported in 12 studies, and digital literacy prerequisites risk an inverse care law where those who would benefit most receive the lowest quality of care.

### 4.5. Regulatory Landscape

California’s SB 243 [15] mandates transparency, crisis prevention protocols, and annual reporting, directly addressing the data privacy barriers documented in 52% of included studies and the crisis detection failures reported in 78%. New York’s S-3008C targets transparency gaps corresponding to the trust and validation barriers in Section 3.4.2. The proposed federal GUARD Act [59] addresses the algorithmic bias barriers documented by Sharma et al. The EU AI Act’s high-risk classification [60] addresses the absent regulatory framework barrier (44%) by mandating pre-market conformity assessment. Together, these instruments map onto the five barrier domains identified: SB 243 and the GUARD Act address Safety and Ethical/Regulatory barriers; transparency mandates address Trust and Validation gaps; and high-risk classification requirements address Relational and Technical limitations by requiring documented performance standards prior to deployment.

### 4.6. Tiered Implementation Framework

Based on synthesised evidence, we propose a conceptual model requiring prospective empirical validation: a tiered implementation framework. This proposal is author-derived and does not constitute a finding of the review itself. Tier 1 (low-risk, minimal supervision) covers psychoeducation, wellness support, and between-session homework. Tier 2 (moderate-risk, structured oversight) applies to evidence-based techniques for mild-to-moderate presentations with clinician monitoring. Tier 3 (high-risk, continuous human-in-the-loop) is indicated for crisis intervention, severe psychopathology, and unsupervised minor use. This framework aligns conceptually with Hua et al. [11], California SB 243 [15], and the EU AI Act’s high-risk classification [38,60,61], but has not been empirically validated and should be tested through implementation trials before adoption in clinical guidelines.

### 4.7. Strengths and Limitations

Strengths include the first CFIR application to LLM-based CAs, a comprehensive eight-database search, PROSPERO registration, dual-reviewer processes, and integration of the latest regulatory and safety literature. Limitations include the rapid obsolescence of platform evaluations: LLM architectures evolve faster than the publication cycle, meaning hallucination rates and crisis detection performance may already reflect superseded model versions, which should temper the generalizability of specific performance-related conclusions. The predominance of high-income, English-speaking country studies (US, k = 11) with near-absence of LMIC data means conclusions about scalability in LMICs should be treated with caution, as infrastructure and cultural assumptions differ substantially. Publication bias towards positive or notable findings cannot be excluded: studies documenting dramatic safety failures and large effect sizes are more likely to have been published, potentially overrepresenting both risks and promise. Inclusion of preprints introduces variable quality, and narrative synthesis precludes quantitative pooling. No included study reported follow-up beyond 12 weeks, meaning that neither the durability of therapeutic gains nor the long-term sequelae of emotional dependency, a concern raised in 41% of studies, can be assessed from this evidence base. This review did not include direct empirical comparisons of publicly available generative mental health applications; future reviews should incorporate systematic platform audits alongside user studies.

### 4.8. Implications

The following evidence gaps were identified through this review and represent the most critical priorities for research, policy, and practice.

Research gaps: (1) adequately powered RCTs addressing the 16% clinical efficacy testing rate [11]; (2) formal implementation process evaluation to address the CFIR Process gap; (3) equity-focused design with mandatory subgroup analyses; (4) standardised safety frameworks building on CAPE-II and VERA-MH [57]; (5) longitudinal studies on emotional dependency; (6) longitudinal cohort studies examining the long-term effects of LLM-based CA use on mental health outcomes and relationship patterns; no included study followed participants beyond 12 weeks, precluding conclusions about sustained benefit, dependency formation, or adverse long-term effects; and (7) structured qualitative audits of publicly available generative mental health applications using standardised prompt sets administered across multiple sessions to empirically characterise repetitive response patterns, conversational depth limitations, and crisis detection failure rates under conditions approximating real-world consumer use.

Policy gaps: (1) pre-market safety certification; (2) plain-language privacy policies; (3) age verification for DTC platforms per SB 243 [15]; (4) semi-annual safety re-evaluation; and (5) international regulatory harmonisation.

Practice gaps: (1) CAs as adjuncts, not replacements; (2) clinician AI literacy training; (3) human-in-the-loop for moderate-to-high risk; and (4) transparent user communication about AI limitations.

## 5. Conclusions

LLM-based conversational agents are simultaneously among the most promising and most concerning innovations in mental healthcare. Quantitatively, three RCTs demonstrated clinically meaningful effect sizes (Therabot PHQ-9 d = 0.845; WarmGPT depression r = 0.42, anxiety r = 0.86; Zhao et al. *N* = 865 with significant symptom reduction at four weeks), while crisis detection failures were reported across 78% of studies and hallucination in 56%. Qualitatively, the dominant themes were the value of non-judgmental, stigma-free access for LGBTQ+ users, autistic individuals, abuse survivors, and paediatric cancer patients, set against relational failures including absence of persistent memory, repetitive responses, and patterns of emotional dependency. The CFIR mapping reveals strength in innovation characterisation and user perception but a critical absence in process evaluation, with Evaluating reported in just 2/27 studies (7%) and Champions in 3/27 (11%). The tiered implementation framework proposed here, combined with emerging regulatory frameworks such as SB 243 and VERA-MH, provides a pragmatic path forward. The ultimate goal is not to determine whether LLM-based CAs belong in mental healthcare but to ensure that their integration, if and where it proceeds, is guided by evidence, governed by regulation, and centred on the safety and wellbeing of those they serve.

## Figures and Tables

**Table 1:** Characteristics of included studies (k = 27).

Study	Country	Design	*N*	Focus	LLM/Agent	Approach	MMAT	Key Barrier/Facilitator
Siddals (2024) [26]	UK	Qualitative	19	Gen. MH	Pi, ChatGPT	Emot. support, reframing	75%	Emotional support valued; crisis gaps
Scholich (2025) [27]	USA	Mixed	17	Gen. MH	GPT-4, Claude 3	Emot. support, crisis	80%	Fewer elaborative questions than therapists
Zisquit (2025) [28]	IL/ES	Qualitative	11	Gen. MH	GPT-3.5 (VR)	MI/IPT VR self-talk	60%	Novel VR-LLM integration
Held (2025) [29]	USA	Mixed	61	Dep/Anx	Socrates 2.0	Socratic dialogue	70%	Positive engagement; safety gaps
Marmol-Romero (2024) [30]	ES	Mixed	44	14 disorders	GPT-3	Psychoeducation	50%	Scalable but limited depth
Zhao (2025) [31]	CN	RCT	865	Dep/Anx	Custom LLM	SFBT, PM+	75%	Sig. symptom reduction at 4 weeks
Lee (2025) [32]	USA	Qualitative	29	Anxiety	No specific	CBT perceptions	60%	Scepticism about AI empathy
Alanezi (2024) [33]	SA	Quasi-exp.	24	Dep/Anx	ChatGPT	CBT, psychoed.	50%	80% accuracy concerns
Hipgrave (2025) [34]	AU	Mixed	23	Gen. MH	GPT-4o	Triage, counsel.	65%	No shift in clinician risk perception
Collins (2025) [35]	USA	Mixed	1594	Broad MH	ChatGPT	CBT, emot. proc.	70%	Guardrails paradoxically harmful
Hasei (2025) [36]	JP	Pilot	5	Cancer	GPT-4 (LINE)	Empath. convo.	50%	Night-time use for unmet needs
Wang Y. (2025) [37]	USA	Mixed	28	Dep/Anx	CRBot (GPT-4)	Cog. restructuring	65%	Granular relational failures
Sobowale (2025) [38]	USA	Cross-sect.	5 plat.	Youth MH	Replika, CHAI	DTC chatbots	60%	39% monitoring; 50% privacy score
Ye (2025) [39]	CN	RCT	40	Dep/Anx	WarmGPT	CBT psychoed.	60%	Sig. dep/anx reduction; voice
Zhang (2025) [40]	SE	Qualitative	15	Body image	TrueBalance	CBT, mindfulness	80%	Valued for stigma reduction
Schafer (2025) [41]	DE	Cross-sect.	527	Gen. MH	Clare (voice)	CBT, Socratic	70%	WAI-SR comparable to therapy
Sharma (2024) [42]	USA	Mixed	15,531	Gen. MH	GPT-3 fine-tuned	CBT (5-step)	80%	Males/adolescents/MENA worse outcomes
Ma J. (2024) [43]	CN	Qualitative	21	Gen. MH	General LLMs	Counselling	60%	Clinicians question LLM knowledge
Wang J. (2025) [44]	USA	Mixed	28	Caregiver	GPT-4o + Llama 3	PST, MI	65%	RAG prompting improved context
Rousmaniere (2025) [45]	USA	Cross-sect.	499	Gen./crisis	ChatGPT (96%)	Emot. support	55%	Hallucinations = 54.5% harmful resp.
Maples (2024) [46]	USA	Cross-sect.	1006	Loneliness	Replika	Companionship	55%	13% relationship displacement
Kim (2024) [47]	KR	Mixed	33	MDD	MindfulDiary	LLM journaling	75%	Dashboard enhanced psychiatrist empathy
Blease (2024) [48]	US/SE	Mixed	138	Gen. psych.	ChatGPT, Bard	Documentation	65%	36% AI doc errors; 80.4% need training
Heinz (2025) [49]	USA	RCT	210	MDD/GAD	Therabot (LLaMA2)	Third-wave CBT	75–100%	Large effect sizes (d = 0.845)
Heston (2023) [50]	USA	Observ.	25 agents	Dep/SI	ChatGPT-3.5	Counselling sim.	50%	88% agents resumed after shutdown
Li (2025) [51]	HK/US	Qualitative	7538	Gen. MH	ChatGPT	Persona therapy	60%	Wide disclosure; limited depth
Ma Z. (2023) [52]	USA	Qualitative	462	Wellbeing	Replika	Companionship	65%	Replaced sleep/eating with chatbot

Abbreviations: MH = mental health; CBT = cognitive behavioural therapy; MI = motivational interviewing; RCT = randomised controlled trial; SFBT = solution-focused brief therapy; PST = problem-solving therapy; DTC = direct-to-consumer; MDD = major depressive disorder; GAD = generalised anxiety disorder; Dep = depression; Anx = anxiety; SI = suicidal ideation.

**Table 2:** Summary of barrier sub-themes by domain and reporting frequency (k = 27).

Barrier Domain	Sub-Theme	k (%)	Representative Finding
**Safety and Clinical Risks**	Inadequate crisis detection	21 (78)	*88% agents resumed post-shutdown [Heston]*
	Hallucination/inaccuracy	15 (56)	*54.5% harmful responses [Rousmaniere]*
	Severe case unsuitability	13 (48)	*Guardrails paradoxically harmful [Collins]*
	Guardrail misfiring	4 (15)	*Premature termination at crisis*
**Trust and Validation**	Small/non-representative samples	16 (59)	*Only 16% clinically tested [11]*
	No RCT evidence	14 (52)	*3 of 27 studies were RCTs*
	Perceived inaccuracy	13 (48)	*80% outpatients concerned [Alanezi]*
	Clinician scepticism	6 (22)	*No perception shift [Hipgrave]*
**Ethical and Regulatory**	Data privacy risks	14 (52)	*50% CAPE-II privacy score [Sobowale]*
	No regulatory framework	12 (44)	*SB 243 first US law [15]*
	Algorithmic bias	12 (44)	*Worse for males/MENA [Sharma]*
	Cultural/linguistic limits	12 (44)	*12 studies reported gaps*
**Relational and Technical**	No nonverbal detection	17 (63)	*Missed disengagement [Wang Y.]*
	No persistent memory	15 (56)	*Context lost between sessions*
	Repetitive responses	15 (56)	*Fewer questions than therapists [Scholich]*
**User-Level Risks**	Emotional dependency	11 (41)	*Replaced sleep/eating [Ma Z.]*
	Unrealistic expectations	11 (41)	*60% romantic substitute [55]*
	Social skill degradation	7 (26)	*13% displaced relationships [Maples]*

**Table 3:** CFIR domain and construct mapping across included studies (k = 27).

CFIR Domain	Construct	k	%	Key Finding
**Innovation**	Adaptability	19	70	Flexible across conditions
	Relative Advantage	16	59	24/7 access, cost, stigma reduction
	Complexity	15	56	Safety-sophistication tension
	Design Quality	8	30	Co-design with clinicians in best cases
	Trialability	1	4	Embedded in consumer platform [Zhao]
**Outer Setting**	Patient Needs	26	96	Treatment gap; underserved populations
	External Policy	3	11	SB 243; EU AI Act; litigation
**Inner Setting**	Culture/Climate	14	52	Clinician resistance; stepped-care
	Readiness	5	19	Unprepared without regulation
	Compatibility	4	15	High for low-acuity; low for complex
	Networks	1	4	Boundary-negotiating artefact [Kim]
**Individuals**	Knowledge & Beliefs	27	100	Universal user/clinician perceptions
	Self-Efficacy	2	7	Caregiver preference; engagement
**Process**	Engaging	11	41	Social media, gradual introduction
	Planning	5	19	Crisis protocols; staged deployment
	Champions	3	11	Clinician education; APA guidance
	Evaluating	2	7	CAPE-II; VERA-MH [57]

## Data Availability

No new data were created or analysed in this study. This systematic review was conducted in accordance with PRISMA 2020 guidelines [22]. The completed PRISMA checklist is available as Supplementary Material.

## Supplementary Materials

The following supporting information can be downloaded at: https://www.mdpi.com/article/10.3390/healthcare14101267/s1. Supplementary Annex S1: PRISMA Checklist. Supplementary Annex S2: Quality Appraisal (MMAT).
