# Supplementary material for: Barriers and Facilitators to the Use of Large Language Model-Based Conversational Agents in Mental Healthcare: A Systematic Review
Source: Healthcare (Basel). 2026 May 7;14(10):1267. doi: 10.3390/healthcare14101267 (PMC13206696; doi:10.3390/healthcare14101267)
Supplement: Supplementary file 1 [file healthcare-14-01267-s001.zip › healthcare-4200591-supplementary.pdf]

PRISMA 2020 Checklist

Supplementary Annex S1: PRISMA Checklist

Section and Topic	Item #	Checklist item	Location where item is reported
**TITLE**			
Title	1	Identify the report as a systematic review.	Title page
**ABSTRACT**			
Abstract	2	See the PRISMA 2020 for Abstracts checklist.	Abstract section
**INTRODUCTION**			
Rationale	3	Describe the rationale for the review in the context of existing knowledge.	Introduction, paragraphs 1–3
Objectives	4	Provide an explicit statement of the objective(s) or question(s) the review addresses.	Introduction, last paragraph
**METHODS**			
Eligibility criteria	5	Specify the inclusion and exclusion criteria for the review and how studies were grouped for the syntheses.	Methods: Section 2.1 Eligibility Criteria
Information sources	6	Specify all databases, registers, websites, organisations, reference lists and other sources searched or consulted to identify studies. Specify the date when each source was last searched or consulted.	Methods: Section 2.2 Information Sources and Search Strategy
Search strategy	7	Present the full search strategies for all databases, registers and websites, including any filters and limits used.	Methods: Section 2.2 Information Sources and Search Strategy
Selection process	8	Specify the methods used to decide whether a study met the inclusion criteria of the review, including how many reviewers screened each record and each report retrieved, whether they worked independently, and if applicable, details of automation tools used in the process.	Methods: Section 2.3 Study Selection
Data collection process	9	Specify the methods used to collect data from reports, including how many reviewers collected data from each report, whether they worked independently, any processes for obtaining or confirming data from study investigators, and if applicable, details of automation tools used in the process.	Methods: Section 2.4 Data Extraction and Quality Appraisal
Data items	10a	List and define all outcomes for which data were sought. Specify whether all results that were compatible with each outcome domain in each study were sought (e.g. for all measures, time points, analyses), and if not, the methods used to decide which results to collect.	Methods: Section 2.4 Data Extraction and Quality Appraisal
	10b	List and define all other variables for which data were sought (e.g. participant and intervention characteristics, funding sources). Describe any assumptions made about any missing or unclear information.	Methods: Section 2.4 Data Extraction and Quality Appraisal
Study risk of bias assessment	11	Specify the methods used to assess risk of bias in the included studies, including details of the tool(s) used, how many reviewers assessed each study and whether they worked independently, and if applicable, details of automation tools used in the process.	Methods: Section 2.4 Data Extraction and Quality

Section and Topic	Item #	Checklist item	Location where item is reported
			Appraisal (MMAT)
Effect measures	12	Specify for each outcome the effect measure(s) (e.g. risk ratio, mean difference) used in the synthesis or presentation of results.	Not applicable (narrative synthesis)
Synthesis methods	13a	Describe the processes used to decide which studies were eligible for each synthesis (e.g. tabulating the study intervention characteristics and comparing against the planned groups for each synthesis (item #5)).	Methods: Section 2.5 Data Synthesis
	13b	Describe any methods required to prepare the data for presentation or synthesis, such as handling of missing summary statistics, or data conversions.	Methods: Section 2.5 Data Synthesis
	13c	Describe any methods used to tabulate or visually display results of individual studies and syntheses.	Results: Tables 1–3; Figures 1–2
	13d	Describe any methods used to synthesize results and provide a rationale for the choice(s). If meta-analysis was performed, describe the model(s), method(s) to identify the presence and extent of statistical heterogeneity, and software package(s) used.	Methods: Section 2.5 Data Synthesis (SWiM; directed content analysis guided by CFIR)
	13e	Describe any methods used to explore possible causes of heterogeneity among study results (e.g. subgroup analysis, meta-regression).	Not applicable
	13f	Describe any sensitivity analyses conducted to assess robustness of the synthesized results.	Not applicable
Reporting bias assessment	14	Describe any methods used to assess risk of bias due to missing results in a synthesis (arising from reporting biases).	Not reported
Certainty assessment	15	Describe any methods used to assess certainty (or confidence) in the body of evidence for an outcome.	Methods: Section 2.4 (MMAT); Results: Section 3.3 Quality Appraisal
**RESULTS**			
Study selection	16a	Describe the results of the search and selection process, from the number of records identified in the search to the number of studies included in the review, ideally using a flow diagram.	Results: Section 3.1 Study Selection
	16b	Cite studies that might appear to meet the inclusion criteria, but which were excluded, and explain why they were excluded.	Results: Section 3.1 Study Selection
Study characteristics	17	Cite each included study and present its characteristics.	Results: Table 1
Risk of bias in studies	18	Present assessments of risk of bias for each included study.	Results: Table 1 (MMAT column); Section 3.3 Quality Appraisal
Results of individual studies	19	For all outcomes, present, for each study: (a) summary statistics for each group (where appropriate) and (b) an effect estimate and its precision (e.g. confidence/credible interval), ideally using structured tables or plots.	Results: Tables 1–2; Sections 3.4–3.5
Results of syntheses	20a	For each synthesis, briefly summarise the characteristics and risk of bias among contributing studies.	Results: Section 3.3 Quality Appraisal; Tables 2–3
	20b	Present results of all statistical syntheses conducted. If meta-analysis was done, present for each the summary estimate and its precision	Not applicable

Section and Topic	Item #	Checklist item	Location where item is reported
		(e.g. confidence/credible interval) and measures of statistical heterogeneity. If comparing groups, describe the direction of the effect.	(narrative synthesis)
	20c	Present results of all investigations of possible causes of heterogeneity among study results.	Not applicable
	20d	Present results of all sensitivity analyses conducted to assess the robustness of the synthesized results.	Not applicable
Reporting biases	21	Present assessments of risk of bias due to missing results (arising from reporting biases) for each synthesis assessed.	Not reported
Certainty of evidence	22	Present assessments of certainty (or confidence) in the body of evidence for each outcome assessed.	Results: Section 3.3 Quality Appraisal; Discussion: Section 4.7 Strengths and Limitations
**DISCUSSION**			
Discussion	23a	Provide a general interpretation of the results in the context of other evidence.	Discussion: Section 4.1 Principal Findings
	23b	Discuss any limitations of the evidence included in the review.	Discussion: Section 4.7 Strengths and Limitations
	23c	Discuss any limitations of the review processes used.	Discussion: Section 4.7 Strengths and Limitations
	23d	Discuss implications of the results for practice, policy, and future research.	Discussion: Section 4.8 Implications
**OTHER INFORMATION**			
Registration and protocol	24a	Provide registration information for the review, including register name and registration number, or state that the review was not registered.	Methods (opening paragraph): PROSPERO (CRD42024601264)
	24b	Indicate where the review protocol can be accessed, or state that a protocol was not prepared.	Not reported
	24c	Describe and explain any amendments to information provided at registration or in the protocol.	Not reported
Support	25	Describe sources of financial or non-financial support for the review, and the role of the funders or sponsors in the review.	Funding section
Competing interests	26	Declare any competing interests of review authors.	Conflicts of Interest section
Availability of data, code and other materials	27	Report which of the following are publicly available and where they can be found: template data collection forms; data extracted from included studies; data used for all analyses; analytic code; any other materials used in the review.	Data Availability Statement

From: Page MJ, McKenzie JE, Bossuyt PM, Boutron I, Hoffmann TC, Mulrow CD, et al. The PRISMA 2020 statement: an updated guideline for reporting systematic reviews. BMJ 2021;372:n71. doi: 10.1136/bmj.n71

Supplementary Annex S2: Quality Appraisal (MMAT)

MMAT = Mixed Methods Appraisal Tool. Ratings are estimated based on study-reported information and reflect the proportion of applicable criteria met. COREQ = Consolidated Criteria for Reporting Qualitative Research; STROBE = Strengthening the Reporting of Observational Studies in Epidemiology; SRQR = Standards for Reporting Qualitative Research; CONSORT = Consolidated Standards of Reporting Trials; IRR = inter-rater reliability; ITT = intention-to-treat; COI = conflict of interest; MI = multiple imputation; HCP = healthcare professional.

Study	Study Design	Estimated MMAT Rating	Key Strengths	Key Limitations
Siddals et al. (2024)	Qualitative	75-80% (3-4/5)	Rigorous reflexive thematic analysis; member-checking; expert review	Convenience sampling; sole coder; self-selection bias toward positive experiences; no negative-case analysis
Scholich et al. (2025)	Mixed methods	75-80% (3-4/5)	Robust design; inter-rater reliability for MULTI coding; saturation; direct human-AI comparison; validated coding framework	Small N=17; scripted (non-naturalistic) scenarios; single time-point; US-centric clinician sample; rapid chatbot evolution
Zisquit et al. (2025)	Qualitative	50-60% (2-3/5)	Clinical psychologist oversight; iterative development; pilot testing; applied thematic analysis by two coauthors	Very small N=11; no data saturation; novelty and social desirability biases; no member-checking; no negative case analysis
Held et al. (2025)	Mixed methods	50-75%	Validated instruments (FIM, AIM, IAM, SUS, MAUQ, WAI-SR); consensual thematic analysis with independent coding	Pre-post without control; convenience/self-selected sample; no multiple comparison correction; non-clinical sample
Marmol-Romero et al. (2024)	Mixed methods pilot	25-50%	Hybrid controlled/open dialogue design; NLP linguistic analysis; psychologist oversight	No control group; no validated pre-post clinical outcomes; convenience sample; anonymous survey prevents linkage; small N=44; researcher-reminder-driven engagement
Zhao et al. (2025)	RCT	75% (moderate-high)	Registered (NCT06346496); CONSORT-compliant; adequate sample; validated instruments (PHQ-9, GAD-7, PANAS)	Non-randomised sequential allocation; non-ITT analysis; high differential attrition (37.3% vs 11%); partial blinding only
Lee et al. (2025)	Qualitative	50-75%	Structured rapid qualitative analysis; multiple researchers; triangulation; consensus-based themes	Small N=29; predominantly White female; single healthcare system; rapid rather than full thematic analysis
Alanezi (2024)	Quasi-experimental + qualitative	50-60%	Clear research question; data collection rigor; thematic analysis	No standardised instruments; single researcher; small geographically restricted sample; reflexivity/saturation not discussed
Hipgrave et al. (2025)	Mixed methods	60-75%	Methodological transparency; dual coding with consensus; quantitative-qualitative triangulation; reflexive limitations	Saturation not assessed; demand characteristics; small sample; potential social desirability bias
Collins et al. (2025)	Mixed methods (secondary data)	50-60%	Large sample (N=1,594); systematic extraction; methodological innovation (GPT-4o coding + Ising network)	Single human coder on 50/1,594 posts; no demographics; Reddit-only; selection and self-report biases
Hasei et al. (2025)	Pre-post pilot	25-40%	Multi-site (3 institutions); iterative testing with 30 HCPs; clinician-reviewed safety protocols	N=5; no control group; unvalidated single-item outcomes; qualitative feedback not systematically analysed; ethics consent waived for paediatric population
Wang Y. et al. (2025)	Mixed methods	65-75%	IRB approval; validated screening (PHQ-9, GAD-7); think-aloud with verbatim transcription; MHP-user triangulation	Single modality; non-clinical sample; small expert panel; no comparison condition; no clinical outcome measures
Sobowale et al. (2025)	Cross-sectional observational	70-80%	STROBE-compliant; validated framework (CAPE-II); Krippendorff alpha 0.81; Bonferroni correction; ecological validity	Only most popular chatbot/platform; 2 personas; primarily single-session; rater team skewed to early 20s

Study	Study Design	Estimated MMAT Rating	Key Strengths	Key Limitations
Ye et al. (2025)	RCT	60% (3/5)	Randomisation described; 100% completion; validated instruments (CDI, SCARED, PANAS-C)	No allocation concealment; no blinding; weak comparator (video); small N=40; power 0.78; no long-term follow-up
Zhang et al. (2025)	Qualitative	80% (4/5)	Constructivist paradigm; thematic saturation reported; data triangulation; SRQR guidelines; reflexivity via memos	Small convenience sample; predominantly educated female; single cultural/linguistic context; no negative case analysis
Schafer et al. (2025)	Cross-sectional survey	60% (3/5)	Large sample (N=527); validated instruments (PHQ-4, WAI-SR, UCLA, Mini-SPIN, SWLS); broad demographics	Social media recruitment bias; Western/English-only; no diagnostic data; self-report; COI; high longitudinal attrition (n=21)
Sharma et al. (2024)	Mixed methods (embedded RCTs)	75-80%	Strong randomisation; large ecological sample (N=15,531); subgroup-targeted RCTs; equity analyses	Single-platform recruitment; high dropout (64%); short-term outcomes only; self-report; no standardised clinical instruments
Ma J. et al. (2024)	Qualitative	75%	COREQ-guided; purposive + snowball sampling; data saturation at N=19+2; member checking; audit trail	Single-country; self-selected expert sample; limited transferability
Wang J. et al. (2025)	Mixed methods (within-subject)	50-60%	Randomised counterbalancing; mixed methods triangulation; clinician co-design	Small N=28; single-session; crowdsourced sample; ceiling effects; no standardised scenario control
Rousmaniere et al. (2025)	Cross-sectional survey	50-75%	Adequate sample (N=499); pre-screened participants; attention checks; IRB approval	Self-report; no validated clinical outcomes; non-representative (80% White, English-only, Prolific); no preregistration
Maples et al. (2024)	Cross-sectional + mixed methods	50-75%	Large sample (N=1,006); validated scales; IRR >80%; pre-specified eligibility	Cross-sectional; self-report; convenience/opt-in sample; no clinical outcomes; no pre-post; suicide findings unsolicited
Kim et al. (2024)	Mixed methods field deployment	50-75%	IRB; multi-disciplinary co-design; 4-week naturalistic field study; clinical population; RM-ANOVA for engagement	Single-site; small N=28; adolescent-dominant; primary outcomes out of scope; no control group; demand characteristics
Blease et al. (2024)	Mixed methods survey	50%	Mixed methods integration; quantitative-qualitative design	18% response rate; self-selected AI-interested attendees; sampling bias; low generalisability
Heinz et al. (2025)	RCT	75-100% (3-4/4)	Pre-registered (NCT06013137); appropriate randomisation; <5% attrition; Holm-Bonferroni correction; MI analyses	Unblinded; WLC only; selection bias via Meta Ads; short follow-up (8 weeks); nondeterministic outputs
Heston (2023)	Observational (simulation)	50% (2/4)	Systematic reproducible methodology; two prompt sets (convergent validity); all eligible agents included	Fixed text prompts; single platform/LLM version; single-author; no human subjects; limited generalisability
Li et al. (2025)	Qualitative	75-80%	Inductive thematic analysis with theoretical saturation; multi-researcher coding; iterative consensus	No member-checking; no negative case analysis; Reddit data only
Ma Z. et al. (2023)	Qualitative	60-70%	Two-stage coding; codebook development; data saturation; three independent coders; consensus; privacy protections	Single platform (r/Replika); no member-checking; no reflexivity statement; non-clinical only
